# “I think I could turn and live with animals”

**DOI:** 10.3201/eid1512.000000

**Published:** 2009-12

**Authors:** Polyxeni Potter

**Affiliations:** Centers for Disease Control and Prevention, Atlanta, Georgia, USA

**Keywords:** Art science connection, emerging infectious diseases, art and medicine, Rosa Bonheur, animalière, realism, naturalism, zoonoses, Plowing in Nivernais, about the cover

**Figure:**
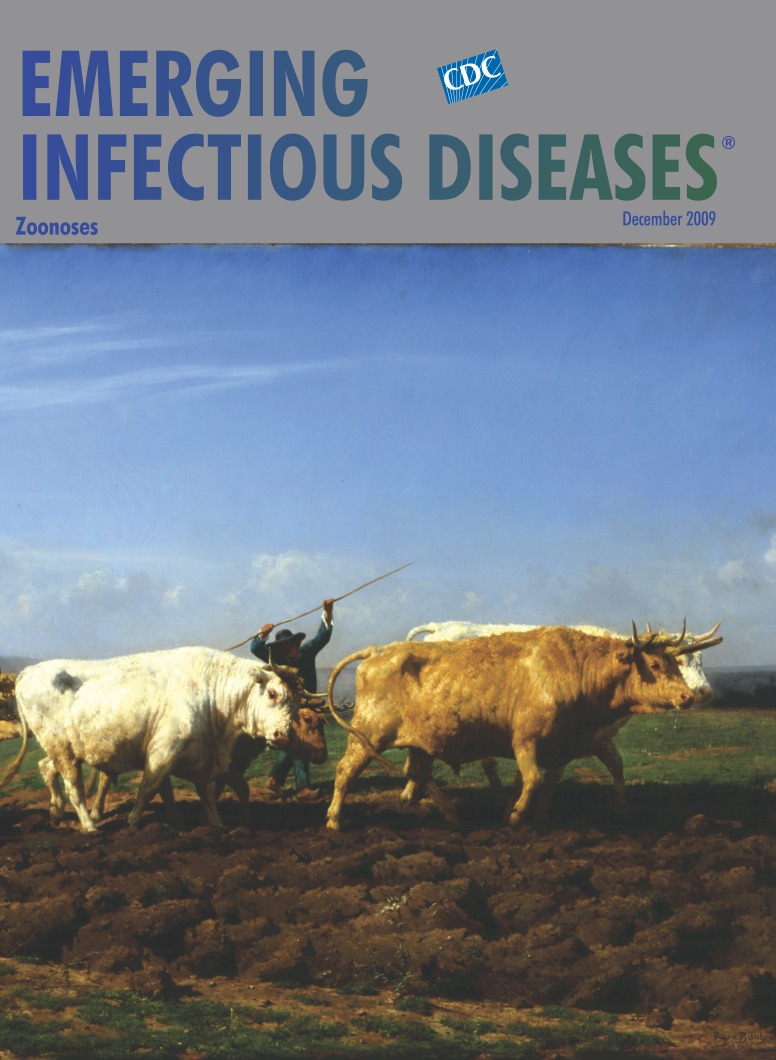
**Rosa Bonheur (1822–1899) Plowing in Nivernais (1850)** Oil on canvas (133.4 cm × 259.1 cm) SN433 Collection of the John and Mable Ringling Museum of Art, the State Art Museum of Florida, a Division of Florida State University

—Walt Whitman

“I wed art. It is my husband―my world―my life-dream―the air I breathe,” said Rosa Bonheur, explaining her life choices. “Art is absorbent―a tyrant. It demands heart, brain, soul, body, the entireness of its votary.” A native of Bordeaux, France, Bonheur was betrothed to art early in life, born into a family of artists. They moved to Paris when she was still a young child. Precocious and headstrong, she had to be coaxed to learn how to read by her mother, who had her select and draw an animal for each letter of the alphabet. Bonheur later attributed her love of drawing animals to this early practice.

During her early days in Paris when the family lived in an apartment, she kept a small menagerie of ducks, rabbits, squirrels, and a sheep that had to be carried up and down the stairs regularly. Expelled from traditional schools for rebelliousness by age 12 and refusing to apprentice with a seamstress, another conventional option, she was turned over to her father Raimond Bonheur, a painter, sculptor, and educator, for instruction. She showed herself a diligent and conscientious art student and soon began copying masterpieces at the Louvre. She assisted him with painting commissions and excelled as sculptor. She started visiting Paris abattoirs and the École nationale vétérinaire d’Alfort to learn animal anatomy by dissecting carcasses. “Oh! You’ve got to be devoted to art to live in pools of blood, surrounded by butchers.”

She also attended horse fairs and farmers’ markets to observe animals’ emotions and behavior. During these outings, “I was forced to recognize that the clothing of my sex was a constant bother. That is why I decided to solicit the authorization to wear men’s clothing from the prefect of police. But the suit I wear is my work attire and nothing else.” Despite her protestations, Bonheur’s independent thinking, original approach to societal restrictions, and bohemian lifestyle created an aura of notoriety about her that at times eclipsed her artistic accomplishments.

She met and became friends with Étienne Geoffroy Saint-Hilaire and his son, Isidore, renowned anatomists and zoologists. Her sympathetic portrayal of animals was influenced by their studies in natural history, particularly the father’s unity of composition principle. He believed that all organisms shared the same underlying design and that diversity in external form was simple variation. If birds and reptiles are built on a single plan, “an accident that befell one of the reptiles … could develop in every part of the body the conditions of the ornithological type.”

Bonheur’s style was part of the realist movement of the mid-1800s, led by Gustave Courbet and Jean-François Millet. In this style, which relied on direct observation and meticulous draftsmanship, naturalism was aligned with social causes and the labor movement. Despite prevailing trends, which favored rural life, the plight of peasants, and the ills of growing industrialization, she chose animals as her subjects. Even in rural scenes, her focus was on them. Her affinity to animals and her devotion to showing them in their natural environment established her as the foremost animalière of her century, one of the best of all time.

Although she was aware of the impressionists, Bonheur did not adopt their style. For inspiration she turned instead to the Parthenon friezes and the romantic paintings of horses by Théodore Géricault and Eugène Delacroix. She was also influenced by her friend, the acclaimed English animal painter Edward Landseer.

Her work attracted early attention. She began exhibiting in the Salon at age 19 and continued to exhibit there successfully over many years. She stopped showing sculpture when she became aware of her brother Isidore’s talent in that art. She “did not want to hinder [his] artistic career.” Her painting Cows and Bulls of the Cantal received a gold medal at the Salon. After this success she received a commission from the government to create a painting of animals at work in the fields. The work, Plowing in Nivernais, was very well received.

When her father died, she succeeded him as director of the art school for girls where he had worked. At that time she also established a studio with friend and fellow artist Nathalie Micas and began to work on massive paintings of horses. One of these, The Horse Fair, became a sensation and attracted the attention of Britain’s Queen Victoria. Bonheur’s fame was far-reaching. She received the Cross of San Carlos of Mexico; membership in the Académie des Beaux-Arts of Antwerp, Belgium; the Commander’s Cross of the Royal Order of Isabella from Spain’s Alphonso XII; and the French Legion of Honor. She handled fame gracefully and wore the medals.

As her commercial success increased, she was able to move to a chateau outside Paris near the forest of Fontainebleau. On these spacious grounds, she created a small zoo, with ponies, deer, monkeys, cattle, and other animals that populated her future work. “One of her pets was a young lion whom she allowed to run about and often romped with.”

Bonheur’s fascination with the New World and the American West began with Buffalo Bill’s Wild West extravaganza, a combination of circus and historical reenactment, during its European tour through France. She traveled to the United States and painted American themes and a famous portrait of Buffalo Bill Cody, who became her friend. Her interest in the United States led to her long connection with Anna Klumpke, an American artist, who after Bonheur’s death, found hundreds of paintings and drawings unseen by anyone in her friend’s studio and pulled together the artist’s (auto)biography.

Plowing in Nivernais, on this month’s cover, is a copy by Bonheur of the original government commission, made a year later and likely inspired by The Devil’s Pool, a novel by George Sand (1804–1876). This story, about the displacement of peasants by industrialization, contained the lines, “But what caught my attention was a truly beautiful sight, a noble subject for a painter. At the far end of the flat plow land, a handsome young man was driving a magnificent team [of] oxen.” Bonheur lived in Nivernais, in central France, for weeks, observing the animals and the land, the people at work, the agricultural tools. Her depiction was so accurate that the region was immediately identified when the painting was unveiled.

“Oxen that rattle the yoke and chain or halt in the leafy shade, what is that you express in your eyes?” wondered Walt Whitman in his poem Song of Myself, “It seems to me more than all the print I have read in my life.” In Bonheur’s own “poem,” the massive beasts crisscross the good earth, plowing uphill. Slow and solid, they dominate a landscape vast as the sky, tails whisking, mucus glistening. The peasants are in the sidelines. Small and unimposing, they follow or step next to them, light-footed, like dancers. The man toward the front holds a thin stick above his head, exerting dominance, choreographing the animals’ movements. He is their colleague, a member of the herd, who grooms and feeds them and lives with them. The animal in the lead squints ahead, one that follows looks directly at us. Industrialization has not yet arrived in this agrarian corner.

Nineteenth-century concerns about the spread of urbanization have only grown in our times. The percentage of residents in urban areas is projected to increase from 50% in 2008 to 70% in 2025. But along with displacement of agriculture, other fears cloud the horizon. Crowded urban areas have become uniquely vulnerable to public health crises, not least of them pandemic (H1N1) 2009. Recent outbreaks in Mexico City and New York demonstrate that surveillance efforts and management of public health communication and response demand exceptional alertness and coordination.

And the animals? “[T]hey are so placid and self contan’d, / I stand and look at them long and long,” wrote Whitman when he offered to “turn and live with” them. For the poet, they held as much fascination as for Bonheur, who first brought them into her apartment and later established them in her own zoo at Fontainebleau, shunning portraiture and trendy interiors to paint them exclusively. He was attracted to their non-humanness, “They do not sweat and whine about their condition, / They do not lie awake in the dark and weep for their sins.” Furthermore, “Not one is dissatisfied, not one is demented with the mania of owning things, / Not one kneels to another, nor to his kind that lived thousands of years ago, / Not one is respectable or unhappy over the earth.”

Bonheur understood animals. Her unconventional spirit was drawn to their wildness. “I too am not a bit tamed, I too am untranslatable, / I sound my barbaric yawp over the roofs of the world.” But, like the poet, she did not know all we share with them. Her furry friends in the apartment or the chateau―rodents, rabbits, goats, deer, cattle, lions―and some that she did not collect, appear in the pages of this issue. For they share with us not just living space but countless infections.

How could the artist have known the litany of zoonoses that continue to complicate our relationship with animals: highly pathogenic influenza A virus (H5N1) in backyard chickens; human trichinellosis associated with ingestion of soft-shelled turtles; new adenovirus in bats; polycystic echinococcosis in jaguar hunters; *Bartonella rochalimae* in raccoons, coyotes, and red foxes; *Ehrlichia chaffeensis* in Sika Deer; *Mycobacterium bovis* and *M*. *tuberculosis* from goats. We have learned to live and work with animals. Now if we could also choreograph the microbes we all share ….
